# Exploiting Micrometer-Scale Replication of Fungal Biotemplates for Multifunctional Uses in Electrochemistry and SERS Substrates

**DOI:** 10.1021/acsomega.4c03431

**Published:** 2024-10-17

**Authors:** Verônica B. Maciel, Adriana M. Fontes, Regina Geris, Zênis N. da Rocha, Jéssica G. S. Ramalho, Antonio F. da Silva, Gabriel C. da Silva, Abdelhafed Taleb, Souad Ammar, Marcos Malta

**Affiliations:** † Institute of Chemistry, Federal University of Bahia, Campus Ondina, Salvador, BA 40110-060, Brazil; ‡ Federal Institute of Bahia, Campus Camaçari, Salvador, BA 40110-060, Brazil; § Institute of Physics, Federal University of Bahia, Campus Ondina, Salvador, BA 40110-060, Brazil; ∥ Institute of Health Sciences, Federal University of Bahia, Campus Canela, Salvador, BA 40110-060, Brazil; ⊥ Department of Chemistry, Federal University of Viçosa, Viçosa, MG 36570-900, Brazil; # Sorbonne Université, 4, place Jussieu, Paris 75321, France; ∇ Laboratory of Interfaces, Treatment, Organization and Dynamics of Surfaces (ITODYS), CNRS, University of Paris Cité, Paris 75005, France

## Abstract

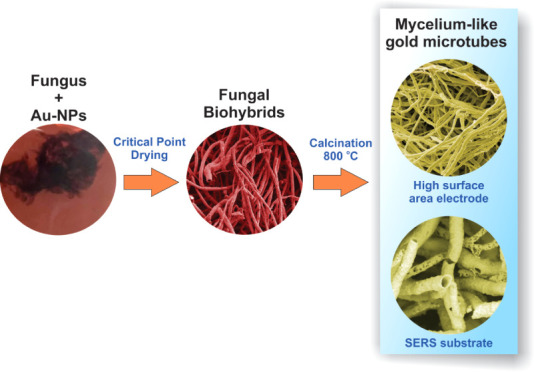

In this paper, filamentous fungi have been used as biotemplates to integrate gold nanoparticles (Au-NPs) into the cell wall. A new chemical mechanism has been proposed to elucidate the assimilation of Au-NPs by fungi, considering the ionic current that arises in the function of fungal metabolism. After biological components were eliminated, mycelium-like gold microtubes have been obtained using different fungal species as precursors. Mycelium-like gold microtubes replicate the biological shape of fungi, presenting inherent multifunctionality. This work presents two promising applications for this material: high surface area electrodes for electrochemical experiments and substrates for SERS detection of organic molecules such as Rhodamine 6G.

## 1 Introduction

Human creativity has shaped Earth by constructing complex structures, such as monuments, buildings, and cities. These structures are remarkable examples of human ingenuity achieved by manipulation of different materials. However, at the nanometer to micrometer scale (10^–9^–10^–6^ m), researchers face difficulties in replicating such complexity of the macroworld, despite the enormous applicative potential. Indeed, inorganic materials that exhibit morphological complexity, such as curves, porosity, and spirals, inherently possess multifunctional properties.^1^ For instance, the silica exoskeleton of unicellular diatoms demonstrates mechanical strength, solar light transparency, and controllable porosity to permit access to nutrients.^2^ With such intricate forms, they hold significant potential as catalysts,^3^ photonic crystals,^4^ and electrochemical energy conversion substrates.^5^

A refined way to design complex inorganic materials relies on organizing nanostructures into predefined biotemplates available in Nature.^6^ In this strategy, interactions between nanoparticles (NPs) and biological surfaces (through variable range forces, such as hydrophobic interactions, van der Waals strengths, and electrostatic forces) organize NPs in larger ensembles, leading to multiscale architected biohybrid superstructures. Unlike isolated NPs, biohybrid superstructures present new collective physicochemical properties besides the possibility of direct integration into functional devices.^7^ Biosystems based on viruses,^8^ bacteria,^9^ algae,^10^ and fungi^11^ have been routinely used as templates for constructing complex tridimensional inorganic structures. In all cases, it takes advantage of utilizing these biological templates regarding their capability of molecular recognition, regular size, wide availability, and relatively low cost.^12^

Filamentous fungi are morphologically complex microorganisms whose elementary growth structure consists of a tubular filament known as a hypha. They are recognized as excellent biotemplates for producing materials with unique morphologies, thanks to their distinctive characteristics such as ease of cultivation, cost-effectiveness, and compatibility with a wide range of materials.^13^ Fungal hyphae are tubular structures surrounded by a rigid cell wall, which protects and maintains cell integrity and viability.^14^ Mycelium, on the other hand, is a root-like structure composed of an interconnected network of branch-like hyphae. The size and shape of these hyphae can vary depending on the fungal species and environmental conditions, allowing the design of various biohybrid architectures. In recent years, several reports have described the ability of fungi to acquire a metallic layer during their growth in media rich in metal NPs, showing new physicochemical properties such as catalytic activity and electrical conductivity.^15^ However, an obvious question concerning using these living organisms as biotemplates is how to control the deposition process of inorganic components. Specifically, there are a few examples of “fine” control of the deposition of metal NPs on the wall of the microorganism surface. Since microorganisms are dynamic biosystems, NPs self-assembly should vary according to the physicochemical properties of the medium, such as nutrients availability, stabilizing agent nature, pH, ionic strength, and metabolites production.^16^ Prior investigations have revealed that Au-NPs assembling on filamentous fungi are particularly sensitive to the concentration of citrate ions.^17^ Filamentous fungi cultivated in low ionic strength solutions present nearly isolated Au-NPs entrapped on the microbial cell wall. In contrast, those grown in high ionic strength solutions evidence aggregated Au-NPs, ultimately forming a dense metallic layer on the biotemplate.

**Figure 1:**
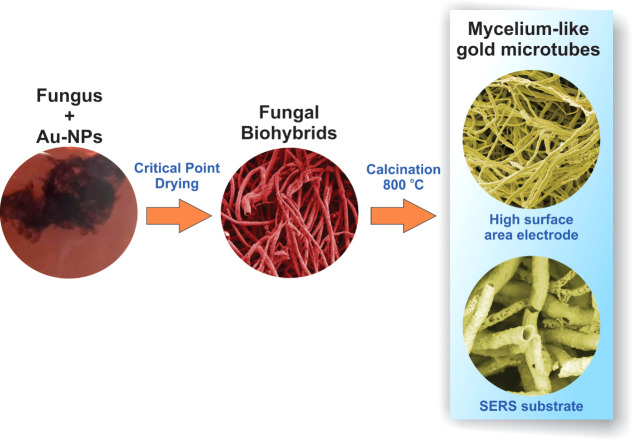
Schematic representation of the formation of mycelium-like gold microtubes. Initially, the fungi are cultivated in citrate-stabilized Au-NPs. Then, biohybrid material is harvested and dried using critical point drying to preserve its tubular morphology. In the last step, calcination of the fungus/Au-NPs biohybrid at 800 °C results in the formation of multifunctional mycelium-like gold microtubes.

Playing with these findings, freestanding gold microtubes that closely replicated the fungal structures have been produced after judicious postheating treatment.^17^ The calcinated fungal biotemplates exhibit a 3D porous interconnected framework with a higher void volume, resembling a mycelium morphology. Thus, as presented in Figure 1, mycelium-like gold microtubes can present promising applications in electrochemistry and surface-enhanced Raman scattering (SERS) sensing.^18^

In this context, this paper aims to continue investigating the applicative potential of mycelium-like gold microtubes, focusing on two applications, electrocatalysis and SERS sensing, with a particular emphasis on the mechanism of fungal/Au-NPs biosystem construction. A chemical mechanism is proposed to contribute to understanding how abiotic Au-NPs are attached and organized on the fungal cell wall. Indeed, the exact mechanism of Au-NPs (or any other nanomaterials) arrangement along the fungal filaments has not yet been elucidated. Some authors suggested that the adhesion of Au-NPs to fungal cells may result from entropic contributions coming from hydrophobic effects, as both the hyphae and NPs display hydrophobic characteristics.^19^ Others claimed that the accumulation of NPs on growing biological cells could be attributed to an as-yet-undiscovered fundamental mechanism that protects the organism against nanomaterial contamination.^20^ Thus, rather than existing as isolated nanoparticles, these contaminants are removed from the surrounding media and “stored” in bulk form on the cell walls. The discussions are still ongoing, and additional studies, such as the present one, would help. Finally, the choice of Au-NPs for this work was motivated by the notorious biocompatibility of these particles, their strong surface-plasmon-resonance band, and chemical stability.^21^ Integrating Au-NPs into filamentous fungi presents real opportunities for producing metallic materials with intriguing tubular structures, which is not trivial at all, particularly when chemical approaches are employed. Compared to other techniques for organizing nanomaterials,^22^ this method is also a simple and economical way to self-assemble nanoparticles around living biosystems. This methodology is particularly suitable for assembling plasmonic nanoparticles in a simple, direct, and one-step process without postfunctionalization. In addition, it allows for the use of different species of fungi, each with unique intrinsic morphological characteristics.

## 2 Experimental Section

### 2.1 Cautions

Autoclaved deionized water (Millipore Milli-Q Water System) was used for synthesizing Au-NPs and cultivating microorganisms. Before experiments, all glassware was thoroughly cleaned with aqua regia solution (3:1 HCl/HNO_3_), rinsed with deionized water, and autoclaved. The fungi species used in this work were *Aspergillus niger* (ATCC, acquired from the André Tosello Foundation), *Phialomyces macrosporus* (isolated from decomposing leaves of the *Manilkara salmani*), *Trichoderma* sp., *Penicillium* sp., and *Talaromyces pinophilus* (generously provided by Muséum National d’Histoire Naturelle, Paris – France).

### 2.2 Synthesis of Au-NPs

Au-NPs were synthesized through a well-described chemical reduction method in water.^17,23^ Succinctly, 1.1 mL (0.135 mmol) of a 0.1 mol·L^–1^ stock solution of HAuCl_4_ (99.9999%, Aldrich) was added and mixed for 1 min in a boiled 500 mL of deionized water. Subsequently, 1.1 mL of a solution containing 1.0% sodium citrate dihydrate (99%, Aldrich) and 0.05% citric acid monohydrate (99%, Aldrich) was added and mixed for 20 s. Finally, a fresh 0.05% sodium borohydride solution (Aldrich) containing 1% sodium citrate and 0.05% citric acid was added. The whole mixture was boiled for an additional 10 min and then cooled to room temperature. Additional solutions with higher citrate ion content can be prepared in parallel by adding 1% sodium citrate and 0.05% citric acid solution aliquots to this colloidal solution. Finally, 50 mL of these Au-NP solutions were dispensed into 125 mL Erlenmeyer flasks and used for cultivating selected fungi.

### 2.3 Microorganisms Culture

Fungal species were cultivated for 7 days in Petri dishes containing 20 mL of a homemade PDA culture medium (PDA = 30% potato, 2.0% dextrose, and 1.5% agar). Then, small pieces of mycelium were carefully separated and incubated in a solution containing 1.0% sodium citrate and 0.05% citric acid for one month. After this period, small mycelium fragments were transferred to citrate-stabilized Au-NP solutions and left to incubate undisturbed for two months under dark and ambient conditions.

### 2.4 Synthesis of Mycelia-Like Gold Microtubes

After microbial growth, the fungus/Au-NP biohybrids were carefully harvested and subjected to critical point drying (CPD). CPD is crucial for preserving the delicate mycelial morphology and preventing deformation or collapse of the intricate 3D structure caused by surface tension effects. To prepare the biohybrid materials for the CPD, fungal mycelia were maintained submerged in solvent during all steps of preparation. First, the gold colloidal solution was replaced with deionized water until the liquid became colorless. Subsequently, we gradually substituted water by adding acetone aliquots. Starting with 30% volume fraction of acetone, the mycelium was immersed in this solution for 15 min. Additional aliquots were introduced at 15 min intervals, progressively increasing the volume fraction to 50%, 75%, and 100%. Ultimately, the mycelial tissue was transferred to a sample holder immersed in dry acetone for ten min before undergoing CPD procedures using a Leica EM CPD 030 instrument.

In contrast to a previous study,^17^ mycelium-like gold microtubes were produced in a quartz furnace, heating fungal biohybrids in an N_2_ atmosphere at 800 °C, with a heating rate of 10 °C·min^–1^ for 2 h. Subsequently, the samples were exposed to a synthetic air atmosphere for 2 h more. To ensure the removal of any residual ash or burning residues, the products were immersed in a 1.0 mol·L^–1^ HCl for 1 h. Afterward, the mycelium-like gold microtubes were transferred to a piranha solution heated to 60 °C. The final materials were thoroughly rinsed with water and dried under a vacuum in a desiccator.

### 2.5 Material Characterization

UV–vis absorption spectroscopy was conducted on a PerkinElmer UV Winlab. Thermogravimetric analysis was performed in a simultaneous TG/DTA system from Labsys Evo from SETARAM. The investigated materials were heated up to 800 °C (10 °C.min^–1^) in flow of synthetic air flow of 100 mL·min^–1^, using platinum crucibles as support for the thermobalance. Scanning electron microscopies (SEM) were carried out on a ZEISS SUPRA 40 (at ITODYS, *Université Paris Cité*) or a JEOL JSM5400 (at LAMUME, *Universidade Federal da Bahia*). Transmission electron microscopy (TEM) analyses were conducted on a JEOL EM 1230 (at FIOCRUZ, Salvador). The preparation protocol for ultrathin sections of fungus/Au-NP biohybrids for TEM analysis was described in detail elsewhere.^17^

### 2.6 Electrochemistry Experiments

Electrochemistry experiments were conducted using a standard three-electrode setup, utilizing an EG&G Princeton Applied Research 273A Potentiostat/Galvanostat. The reference electrode was an Ag/AgCl reference electrode, and the counter-electrode was a platinum wire. The working electrode was prepared as follows: a small piece of mycelium-like replica was gently pressed against Carbon Vulcan-mineral oil paste and then mounted onto the surface of a graphite electrode. Cyclic voltammetry measurements were carried out to assess the electroactive response of the mycelium-like gold microtubes. These experiments utilized a 9.97 × 10^–4^ mol L^–1^ aqueous solution of K_3_[Fe(CN_)6_]^3–^ in a 1.0 mol·L^–1^ H_2_SO_4_ aqueous electrolyte solution. The electrochemical surface area (ECSA) was estimated by integrating the reduction peak related to the surface oxide layer formed by scanning the mycelium-like replica to oxidizing potentials in a 1.0 mol·L^–1^ H_2_SO_4_ solution.

### 2.7 SERS Experiments

A JASCO Raman Spectrometer, model NRS-5100, equipped with a monochromatic laser emitting radiation at 532 nm, was used for SERS analysis of the mycelium-like gold microtubes. In practice, the mycelium-like replica was immersed in 1 mL solution of Rhodamine B (10^–8^ mol L^–1^) for 2 h. They were then transferred to a glass slide and analyzed by using the Raman spectrometer.

## 3 Results and Discussion

### 3.1 Fungi/Au-NPs Biohybrid Assembly Mechanism

**Figure 2:**
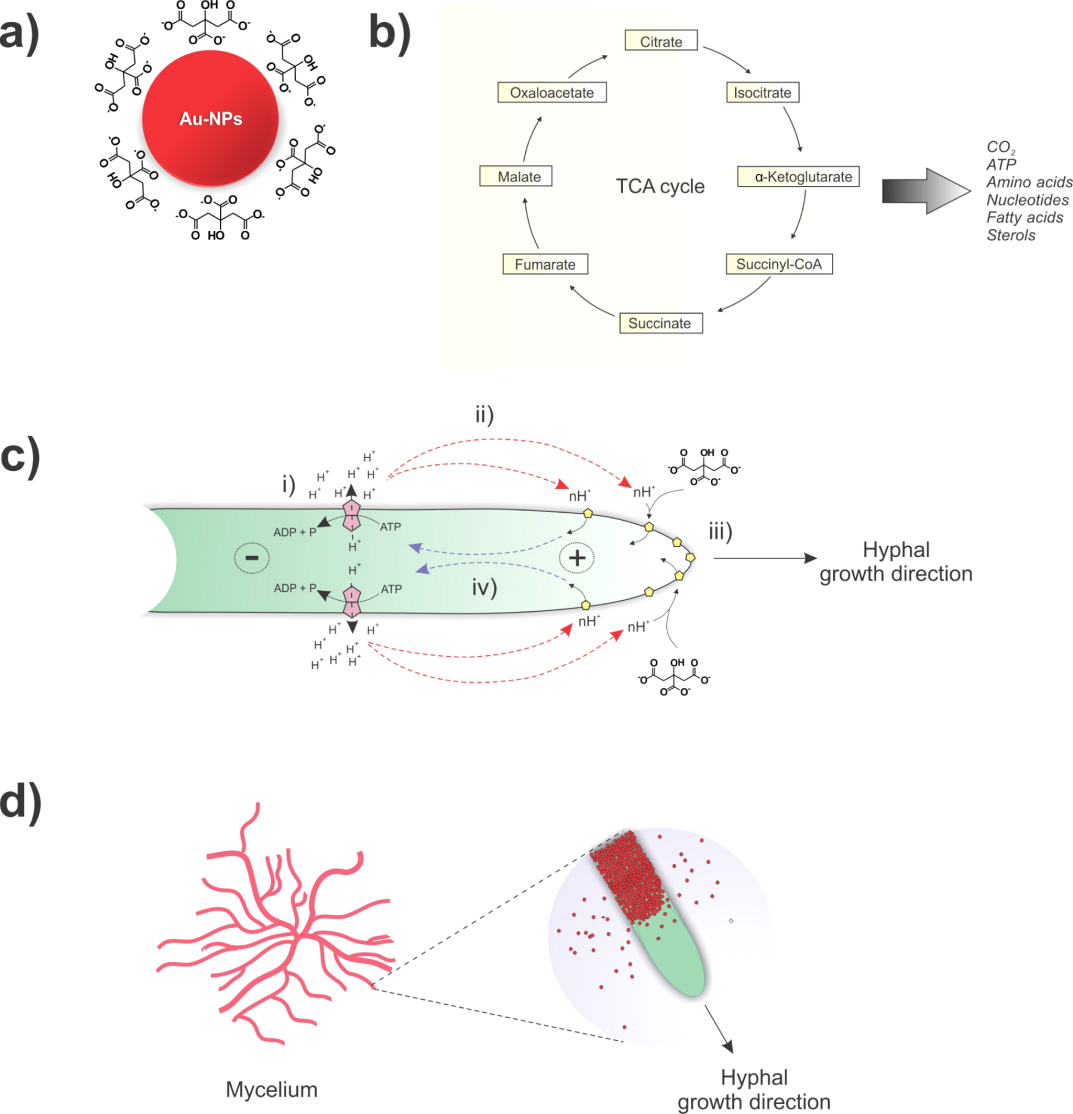
(a) Schematic model of binding of citrate anions on the surface of Au-NP. (b) TCA cycle and some products of fungal anabolism/catabolism. (c) Schematic representation of transcellular ionic current generated during fungal hyphae growth. (d) Negatively charged Au-NPs may be attracted to the hyphal tip during polarized fungal growth, binding to functional groups of cell wall components.

To tentatively elucidate the Au-NPs assembly mechanism, one has to remember that the integration of Au-NPs occurs exclusively in viable cells growing in a solution containing a carbon source.^17,19,23^ The integration of Au-NPs (or any other nanomaterial) to the fungal cell wall occurs during the natural growth process of the mycelium. In simpler terms, as the fungal mycelium grows, it can incorporate nanomaterials like Au-NPs into its cell wall.^24^ It was also established that citrate ion concentration in the cell medium affects the aggregation state of Au-NPs.^19^ For instance, when fungi are cultivated in solutions with lower citrate concentrations, spaced Au-NPs assemble on the cell wall, with additional nanoparticles diffusing and decorating the fungal hyphae in successive layers. In contrast, Au-NPs form a dense metallic layer around the microorganisms at higher citrate concentrations due to reduced interparticle electrostatic repulsion and increased ionic strength. This results in only partial stabilization of the nanoparticles by cell wall components, leading to the formation of agglomerates trapped within the cell wall during fungal growth.^17^ Having that in mind, one may consider that in the present case, citrate ions play a pivotal role in the electrostatic stabilization of Au-NPs since they define the final particle surface charge (Figure 2a). Besides, given the sole availability of citrate as a carbon source, microorganisms necessitate the utilization of the Tricarboxylic Acid Cycle (TCA cycle, Figure 2b) for both anabolic and catabolic processes.^25^ This unique metabolic adaptation enables filamentous fungi to prosper in environments characterized by limited or absent glucose by exploiting citrate as an alternative precursor for essential biomolecule synthesis.^26^ This means that citrate ions may also serve as a vital carbon and energy source for fungal cells. Note that cultivating biohybrids in citrate-stabilized Au-NP colloids took approximately two months because citrate is considered a poor carbon source for fungal growth.

The fungal cell growth occurs through the citrate uptake, an energy-consuming process based on citrate/proton symport mediated by a pH gradient (ΔpH).^27^ Therefore, filamentous fungi do not directly utilize their adenosine triphosphate (ATP) nucleotides for nutrient uptake; rather, ATP hydrolysis by ATPases facilitates proton extrusion across the cell membrane, thus establishing a transmembrane pH gradient, as illustrated in Figure 2ci.^28^ Because protons are expelled in the subapical regions of the hyphae and transverse fungal cells, they travel to areas close to the apex driven by their electrochemical gradient, as illustrated in Figure 2cii (see red arrows). This flow of protons is sent inward to the fungal cell by porters located at the hyphae tip, enabling nutrient uptake via citrate-porter-H^+^ complexes (Figure 2ciii).^28,29^ Finally, protons flow through the cytoplasm along the length of hyphae, as shown in Figure 1civ (see blue arrows) and exit at a distinct location on the cell wall surface, where outward current predominates.

As depicted in Figure 2c, the acquisition of citrate ions mediated by protons establishes a well-defined circuit for the circulation of the ionic current around the fungal cell. Thus, during fungal growth in environmental conditions, i.e., in the absence of NPs, the electric field generated by ionic currents may induce the migration of cytoplasmic constituents and organelles toward the apex of the fungal hyphae.^29,30^ Note that extensive measurements performed on *Neurospora crassa* fungus, using vibrating electrode techniques, provided evidence of electricity attributed to the flux of protons through extracellular currents.^29,31,32^

A significant yet overlooked aspect of integrating nanomaterials into filamentous fungi is the presence of transcellular electric current, resulting in the generation of an external electric field carried by H^+^. The proton circulation at the fungal tip renders it acidic and electropositive, relative to outward current areas. Our hypothesis suggests that electric fields generated by these ionic currents are the main driving force that attracts negatively charged Au-NPs (and other negatively charged NPs), causing their accumulation near the fungal apex (Figure 2d). Thus, the electrostatic attraction of Au-NPs by the fungal surface can be propelled by the electric fields at the polarized growth tip of the hyphae. Experimental evidence of the essential role of transcellular electric current in integrating NPs is further supported by three observations: first, fungal cells do not accumulate NPs if they are not grown (i.e., during the acquisition of the nutrients);^19^ second, the accidental internalization of NPs into the cytoplasm and fungal organelles only occur during the acquisition, transport, and assimilation of nutrients from the environment;^23,33^ finally, when fungi are exposed to an exogenous electrical field, growth is redirected toward the anode or cathode (depending on the species studied), a phenomenon called galvanotropism.^31^ Therefore, as the growth of the microorganism is polarized by electric fields, we can conclude that charged particles in solution are attracted to the fungal hyphae as a function of the transcellular electric current.

In summary, the simple assimilation of citrate ions by fungi, followed by their subsequent depletion near Au-NPs, is not the principal determinant behind the integration of NPs into fungal structures. Instead, the significant factor lies in “ionic currents” traversing fungal cells. In essence, this proposition accentuates the concept that negatively charged Au-NPs are attracted to the apex of filamentous fungi. Consequently, when near the emerging fungal wall, Au-NPs predominantly interact electrostatically with the charged functional groups of the cell wall biopolymers. Contrary to earlier studies, considering a circulating ion current elucidates that other materials could also be influenced by the fungal electric field, potentially leading to their attraction to the cell surface, where they bind and form an artificial layer around the cell. This phenomenon was consistently observed during the growth of various fungal species, specifically during the nutrient acquisition process.

### 3.2 Fungi/Au-NP Biohybrids Characterization

**Figure 3:**
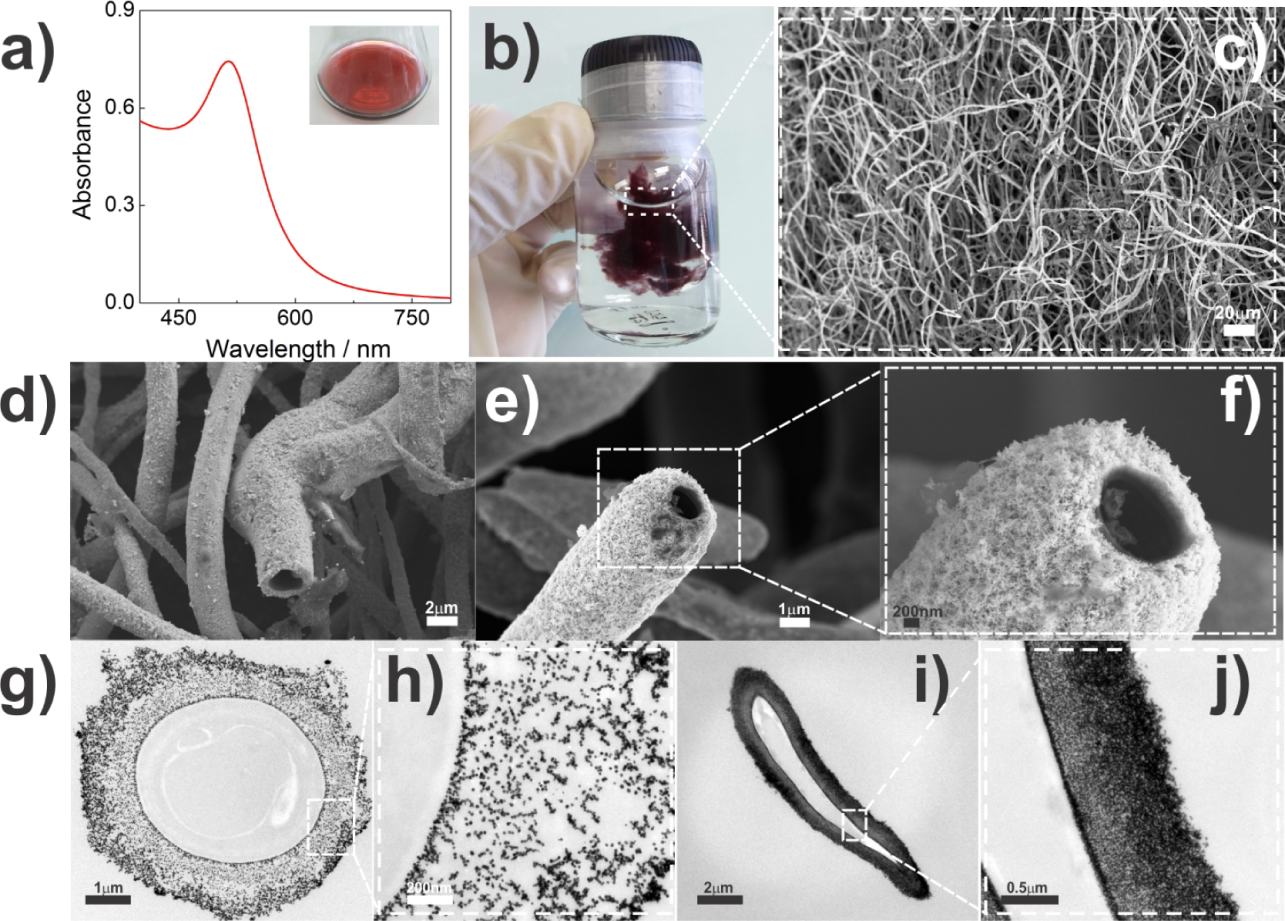
(a) UV–vis spectrum of a citrate-stabilized Au-NPs solution. Inset: fresh colloidal solution of Au-NPs used in fungal cultivation. (b) Digital image of the mycelial tissue *ofTalaromyces pinophilus*/Au-NPs immersed in deionized water. (c) and (d) SEM micrographs of *Aspergillus niger*/Au-NPs biohybrid. (e) and (f) SEM micrographs of an isolated *Phiolomyces macrosporus*/Au-NPs biohybrid filament and its tip. (g) and (h) TEM micrographs of the transversal cross-sectioned region of an *Aspergillus niger* hypha after Au-NPs incorporation. (i) and (j) TEM micrographs of the longitudinal cross-sectioned biohybrid *Trichoderma* sp./Au-NPs.

Before nitrogen pyrolysis, the as-produced biohybrids exhibit a red color due to the assimilation of Au-NPs by the microorganisms (Figure 3b shows a typical image of *Talaromyces pinophilus*/Au-NPs mycelium, dispersed in deionized water), almost the same color than the starting free Au-NPs aqueous colloid (Figure 3a). SEM micrographs collected on the recovered biohybrids clearly illustrate their tubular morphology (Figure 3c–e). These tubular cells exhibit variations in diameter, composition, and cell wall thickness among different species.

The incorporation of Au-NPs into the cell walls of the filamentous fungi was also confirmed by the collected TEM images (Figure 3g–j). Taking *Aspergillus niger* and *Trichodermasp*. species as illustrative examples, the following insights can be derived from the obtained images: Au-NPs with a diameter of around 14.4 nm (Figure S1) can be found almost isolated from each other, indicating their stabilization through a steric effect by the fibrillar biomolecules of the cell wall (Figure 3g). Notably, biopolymers in the cell wall, such as glucans, chitin, and glycoproteins, encapsulate and stabilize the NPs by interacting with their citrate carboxylate and/or surface groups. Structurally, such biopolymers form fibers,^34^ and we hypothesized that the Au-NPs are organized by aligning with the orientation of these biopolymer nanofibers. Another point observed is the robustness of the bond between the NPs and the fungal cells. As exemplified in Figure 3h, the Au-NPs are deeply embedded within the biological matrix of the cell wall, making the release of NPs back into the solution feasible only through the destruction of the fungal wall. Lastly, the self-assembly process of the NPs takes place indiscriminately on the surface of the cell, which is confirmed by the formation of the metal layer uniformly and with regular thickness over the entire surface of the fungal cell.

**Figure 4:**
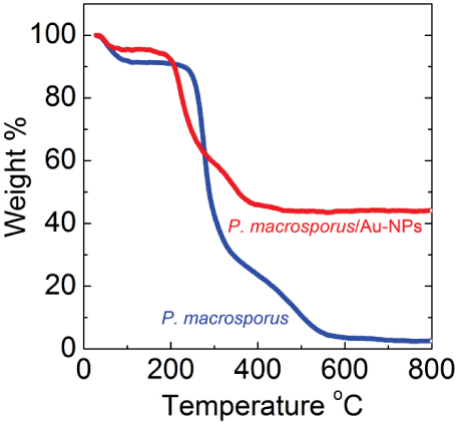
Thermogravimetric analysis of native *P. macrosporus* and the as-produced *P. macrosporus*/Au-NPs biohybrids.

To quantify the assimilation of Au by microorganisms, thermogravimetric analysis was conducted on samples of native fungi and fungus-Au-NPs biohybrids in an oxidizing atmosphere. Although the thermal decomposition of fungal biomass varies from species to species, we used the fungus *P. macrosporus* as a model to exemplify degradation characteristics and the amount of residue after the heating process. Figure 4 illustrates the decomposition of native *P. macrosporus*, exhibiting a total mass loss of 98.1%. When analyzing the derivative thermogravimetric curve (DTG, available in the Supporting Information, Figure S2), three distinct regions of mass loss were identified: first, between 32 and 134 °C, representing the loss of weakly adsorbed water; second, an exothermic event resulting in a significant mass loss between 256 and 408 °C due to the combustion of polysaccharides and proteins; and finally, a final exothermic event between 442 and 544 °C, associated with the combustion of char residue. Thus, from DTG analysis, native *P. macrosporus* showed two exothermic mass losses at 323 and 487 °C, respectively. These findings align with prior research.^17,35^

In contrast, thermogravimetric analysis of the *P. macrosporus*/Au-NPs biohybrid (Figure 4, red line) elucidates the remarkable capability of microorganisms to assimilate metallic particles. Indeed, in the presence of Au-NPs, only 64.0% of the initial mass was lost, and the reduction in mass proceeded mainly at a lower temperature range, between 187 and 409 °C. The maximum rate of degradation of biological components occurs at temperatures of 225 and 352 °C, which is substantially lower than the degradation temperature values observed for the native fungus. Although we do not have a definitive answer for this observation, it is related to the general ability of metallic nanoparticles to catalyze the decomposition of polymers. For instance, Chen and colleagues observed that Pt particles within hydroxyl-terminated generation-4 polyamidoamine dendrimer (Pt-G4OH) catalyze thermal decomposition, allowing the dendrimer to decompose at lower temperatures than the pure G4OH dendrimer.^36^ According to these authors, complete decomposition of the G4OH dendrimer was achieved in air at 500 °C, whereas decomposition of the Pt-G4OH dendrimer was completed at 400 °C, leaving only Pt metal hind.

### 3.3 Mycelium-Like Gold Microtubes Characterization

**Figure 5:**
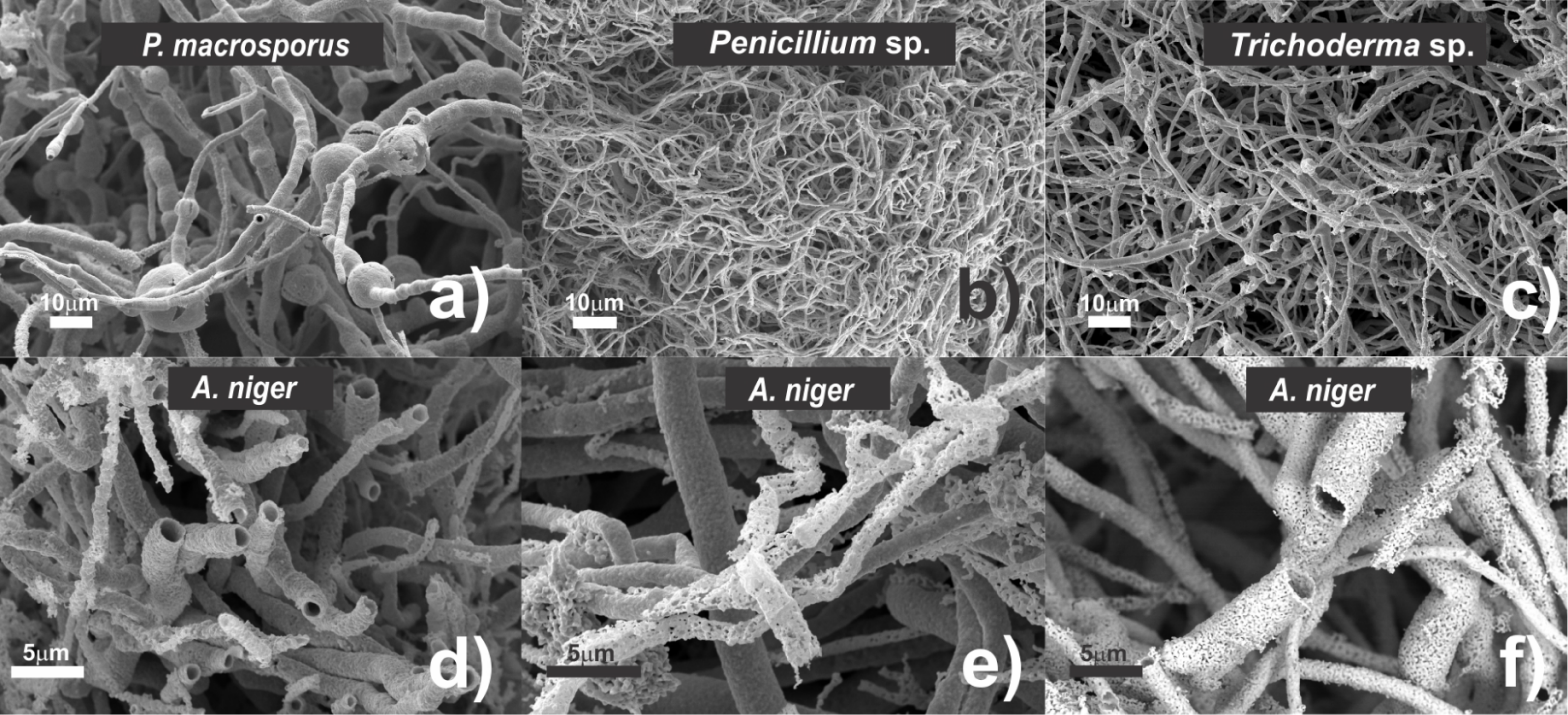
SEM micrographs of mycelium-like microtubes obtained from different fungal species exemplifying the 3D metallic network. a) *Phialomyces macrosporus*, b) *Penicillium*sp. and c) *Trichoderma* sp. replicas. d–f) *Aspergillus niger* replicas.

After calcination, which allowed the elimination of the biological template under nitrogen and synthetic air flux at 800 °C, SEM observation on the resulting matter evidenced a typical structure of mycelium-like microtubes generated (Figure 5). Two notable features become apparent when comparing fungal biohybrids to these mycelium-like replicas. First, the mycelium-like material takes on a distinct golden hue, similar to a small gold nugget. Furthermore, the metallic structure is delicate and requires careful handling. In addition, it is essential to emphasize the contraction of the mycelium-like replica after removing the biological component. Therefore, the thermal treatment induced the coalescence and aggregation of Au-NPs due to the destruction of the stabilizing biopolymers, resulting in a microtubular structure miming the morphology of fungal cells. Figure 5a–c shows the general morphology obtained with the same magnification of the replica of fungal mycelia from three different species: *P. macrosporus*, *Penicillium* sp., and *Trichoderma* sp.

The most important morphological feature of the replicates, as demonstrated by the SEM micrographs of the fungi studied and exemplified by the *Aspergillus niger* replica (Figure 5d–f), is that the resultant mineralized products exhibit lengthy and branched filamentous metallic structures. Whatever the fungal species, their distinct morphologies are almost faithfully replicated (Figure 5), even if the surface of the metallic mycelium replicates exhibits some rough appearance and sometimes small protuberances and irregularities. These irregularities can be observed in detail in the SEM micrographs of *A. niger* different replicas recorded at high magnification, namely, nanopores, rugosities, and occasioned connections between the hyphae, among others (see Figures S3 and S4 for other examples).

Designing materials across multiple length scales, ranging from the nanometric to the micrometric regime, represents a significant technological and scientific endeavor. For example, gold electrodes with complex morphologies offer numerous benefits for electrochemical applications such as enlarged specific surface areas and a multimodal pore structure with void space that permits access to electroactive species. Consequently, these materials facilitate efficient access of reactants to the surface, enhancing the (electro)catalytic process.

### 3.4 Mycelium-Like Gold Microtubes as an Efficient Electrocatalyst

The enlarged specific surface areas and the multimodal pore structure with a void space of the produced gold replicas make them particularly valuable for electrocatalysis. They facilitate reactant access to the catalytic gold surface, enhancing all of the (electro)catalytic processes. As a preliminary study, we focused our investigations on determining the electrochemically active area (ECSA) of the engineered gold replicas in the presence or absence of a redox species. Figure S5 exemplifies the electrode prepared with the replica of the fungal mycelium.

**Figure 6:**
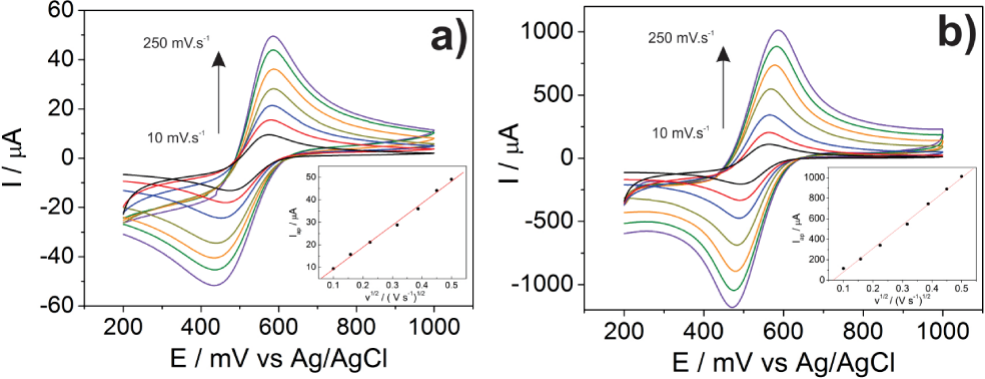
Cyclic voltammograms of Fe(CN)_6_ in a 1.0 mol·L^–1^ H_2_SO_4_ electrolyte recorded using (a) a bulk gold electrode and (b) a mycelium-like gold electrode (*A. niger* replica), for different scan rates (10 to 250 mV·s^–1^). Insets: cathodic peak currents versus square root of scan rates.

The measurements were carried out in a 1.0 mol L^–1^ H_2_SO_4_ aqueous electrolyte, containing first potassium ferrocyanide redox reagent (9.97 × 10^–4^ mol L^–1^). Figure 6 shows the cyclic voltammograms of a flat gold electrode and mycelium-like replica at different scan rates in the 10–250 mV·s^–1^ range. Both electrodes exhibit quasi-reversible voltammograms for the redox reagent, which means that the electron transfer reaction is also diffusion-controlled for the biomimetic metal. Using the Randles-Sevcik equation, the ECSA value, in the presence of the ferrocyanide reagent, was estimated for both electrodes:^37^
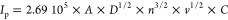
1

where *I*p is the peak current, *A* is the electrochemical area, *D* is the diffusional coefficient, *n* is the number of electrons transferred during the redox reaction, ν is the scan rate, and *C* is the analyte concentration. As shown in the insets of Figure 6a,b, both the peak currents *I*p of bare gold and metallic mycelium-replicas electrodes were proportional to the square root of the scan rate, which permitted obtaining an electroactive surface area of 0.1 cm^2^ and 3.1 cm^2^, respectively, highlighting the positive effect of the engineered microstructure of the gold replicas.

ECSA values of bulk gold and gold replicas were also assessed by analyzing the cyclic voltammograms of the two electrodes in 1.0 mol·L^–1^ H_2_SO_4_ electrolyte in the absence of the ferrocyanide reagent (Figure S6). Both exhibit an anodic peak during forward scans, indicative of oxide layer formation (notably assuming the formation of an oxide monolayer on the metal) and a well-defined cathodic peak in the opposite voltage direction due to the reduction of the oxide layer.^38^ Consequently, by integrating the reduction peak and employing a charge of 390 μC cm^–2^ to reduce a monolayer of gold oxide,^39^ values of 0.03 cm^2^ and 1.35 cm^2^ were determined for flat electrode and mycelium like replica, respectively. Once again, a net increase in the electrochemically active area was observed for the gold replicas. Such high ECSA values confirm the potential of the engineered gold microtubules to be used as efficient electrocatalyst materials for selected redox reactions.

### 3.5 Mycelium-Like Gold Microtubes as an Efficient SERS Substrate

Mycelium-like microtubes were also tested for SERS sensing since they present the required microstructural characteristics for SERS signal enhancement. Developing substrates for SERS is crucial for analyzing biomolecules, contaminants, and disease biomarkers and is fundamental in scientific research and nanotechnology.^40^ Gold replicas exhibit intricate nanostructures with imperfections, such as a rough surface, a high density of pores, curvatures, and aggregated particulate grains, which would help generate intense electromagnetic fields upon illumination with laser light.^41^

**Figure 7:**
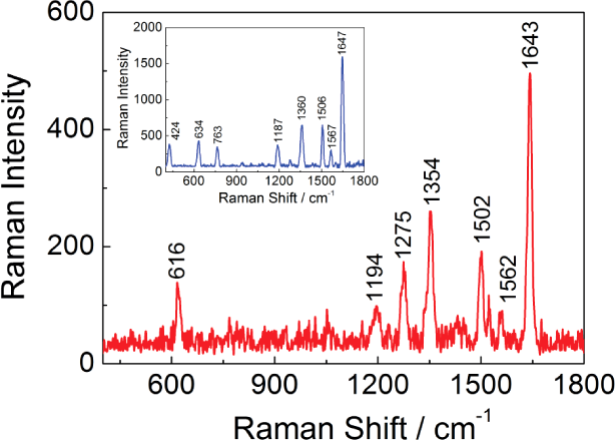
Raman spectra of Rhodamine 6G. SERS effect was observed in mycelium-like microtubes (*P. macrosporus*replica) using 10^–8^ mol L^–1^ Rhodamine 6G solution (red line). Inset: 10^–2^ mol L^–1^ Rhodamine 6G solution droplet on a silicon wafer. Curves were adjusted through baseline remotion to facilitate comparison.

For such a purpose, the Raman spectrum of a concentrated chromophore, Rhodamine 6G (1.0 × 10^–2^ mol L^–1^), was recorded after the deposition of drops of solution in a silicon wafer (inset, Figure 7). It was also recorded after contact with gold replicas. In practice, the metallic mycelium substrate was prepared by immersing the replica piece in a diluted solution of Rhodamine 6G (1 × 10^–8^ mol L^–1^). After 2 h, the replica was separated from the solution and placed directly onto a silicon wafer without the step of drying. Remarkably, a significant enhancement in the Raman signal was observed on the mycelium-like substrate, as presented in Figure 7, confirming the versatility of metallic mycelium toward SERS sensing.

Due to the complexity of the shape of mycelium-like microtubes, we determined that the most direct method for calculating the enhancement factor was to compare the Raman intensities with and without the presence of the SERS substrate. Thus, considering a Rhodamine 6G solution with a concentration *C*_RS_ (1 × 10^–2^ mol L^–1^), which produces a Raman signal *I*_RS_ under non-SERS conditions, and under identical experimental and preparation conditions, the same analyte on the SERS substrate, with a diluted concentration (*C*_SERS_ = 1 × 10^–8^ mol·L^–1^), gives a SERS signal *I*_SERS_. The analytical enhancement factor (AEF) was defined in eq 2:^42^
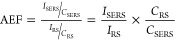
2

Therefore, the AEF found was 1.84 × 10^5^, confirming the versatility of metallic mycelium as a substrate for SERS detection of rhodamine B6.

## 4 Conclusions

In summary, this study presented results regarding the production, characterization, and potential applications of nanostructured gold microtubes designed to replicate the morphology of filamentous fungi. Initially, we proposed a chemical mechanism for incorporating Au-NPs, considering the presence of ionic currents along the tips of fungal hyphae that arise as a result of the development of the microorganism. Consequently, concurrent with the metabolic/anabolic processes involved in hyphal growth, there is an electrostatic attraction that occurs between the negatively charged citrate coated Au-NPs and the polarized hyphal apex. To the best of our knowledge, this mechanism is the first proposal to give a chemical perspective to the assembly of nanomaterials on filamentous fungi, considering the ionic currents produced by fungi during their growth. We demonstrated that upon removal of the cellular components, the resulting metallic mycelium displays multifunctional properties. First, mycelium-like gold microtubes display a surface area 45 times greater compared to conventional electrodes. Second, they serve as substrates for observing surface-enhanced Raman spectroscopy (SERS) effects, enabling the detection of Rhodamine 6G dye at concentrations as low as 10^–8^ mol L^–1^.

## Supporting Information Available

Complementary DTG curves, TEM micrograph, SEM micrographs, and cyclic voltammograms (PDF)
