# Supplementary material for: Exploiting Micrometer-Scale Replication of Fungal Biotemplates for Multifunctional Uses in Electrochemistry and SERS Substrates
Source: ACS Omega. 2024 Oct 17;9(43):43385–94. doi: 10.1021/acsomega.4c03431 (PMC11525492; doi:10.1021/acsomega.4c03431)
Supplement: Supplementary file 1 — ao4c03431_si_001.pdf [file ao4c03431_si_001.pdf]

Supplementary Information

Exploiting Micrometer-Scale Replication of Fungal Biotemplates for Multifunctional Uses in Electrochemistry and SERS Substrates.

Verônica B. Maciel[1,2], Adriana M. Fontes[1], Regina Geris[1], Zênis N. da Rocha[1], Jéssica G. S. Ramalho[3], Antonio F. da Silva[3,4], Gabriel C. da Silva[5], Abdelhafed Taleb[6], Souad Ammar[7] and Marcos Malta[1]*

1 Institute of Chemistry, Federal University of Bahia, Campus Ondina, Salvador (BA), Brazil.

2 Federal Institute of Bahia, Campus Camaçari, Camaçari (BA), Brazil.

3 Institute of Physics, Federal University of Bahia, Campus Ondina, Salvador (BA), Brazil.

4 Institute of Health Sciences, Federal University of Bahia, Campus Canela, Salvador (BA), Brazil.

5 Department of Chemistry, Federal University of Viçosa, Viçosa (MG), Brazil.

6 Sorbonne Université, 4, place Jussieu 75321 Paris - France

7 Laboratory of Interfaces, Treatment, Organization and Dynamics of Surfaces (ITODYS), CNRS, University of Paris Cité, Paris, France

KEYWORDS: fungal biohybrids, bioinspired materials, high surface area electrodes, SERS substrates

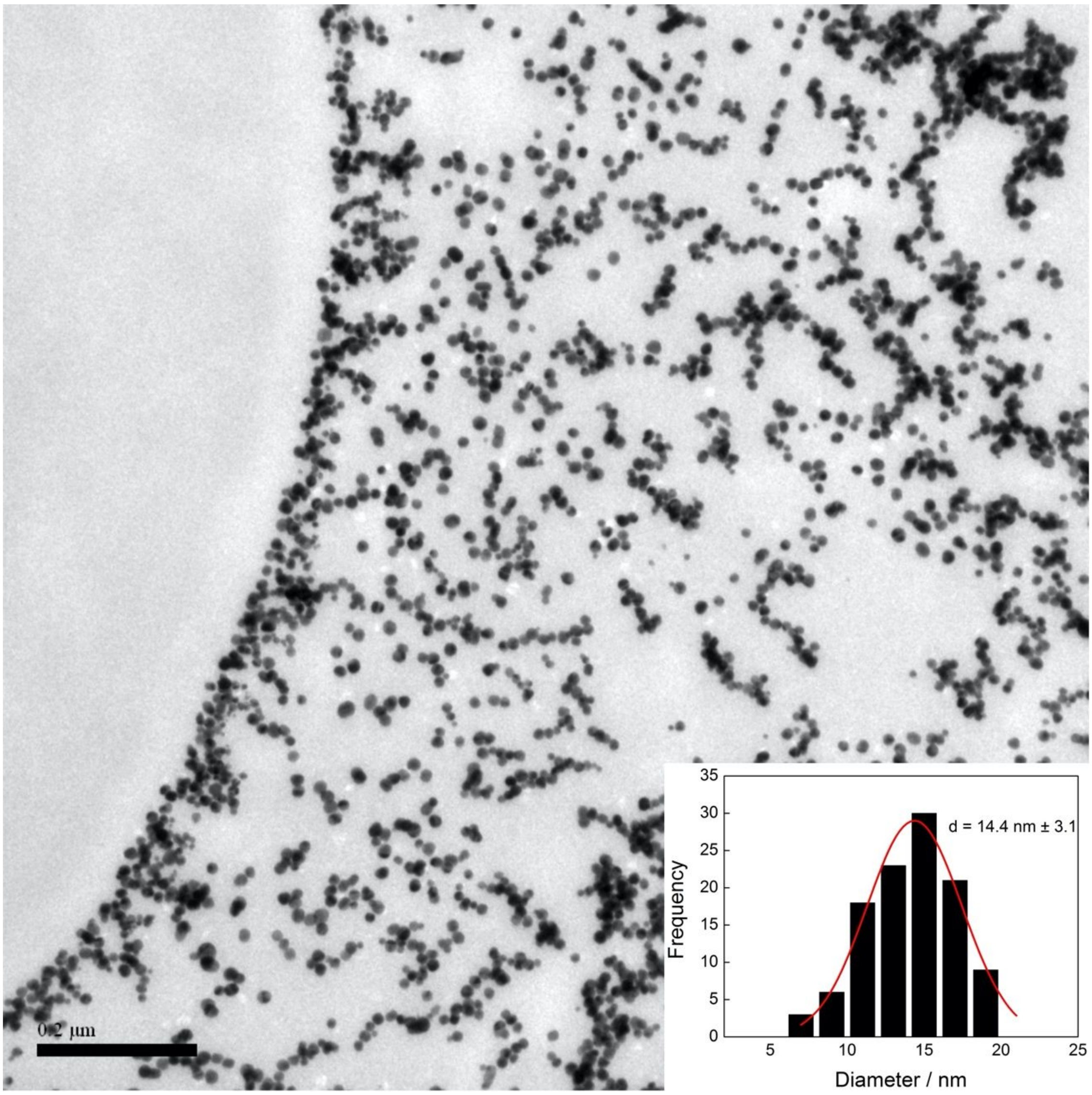

Figure S1: TEM micrograph and particle size histogram of a thin section of Au-NPs embedded at the fungal cell wall. The size distribution histogram was made from 110 isolated particles randomly chosen from the TEM image.

Maciel *et al.*

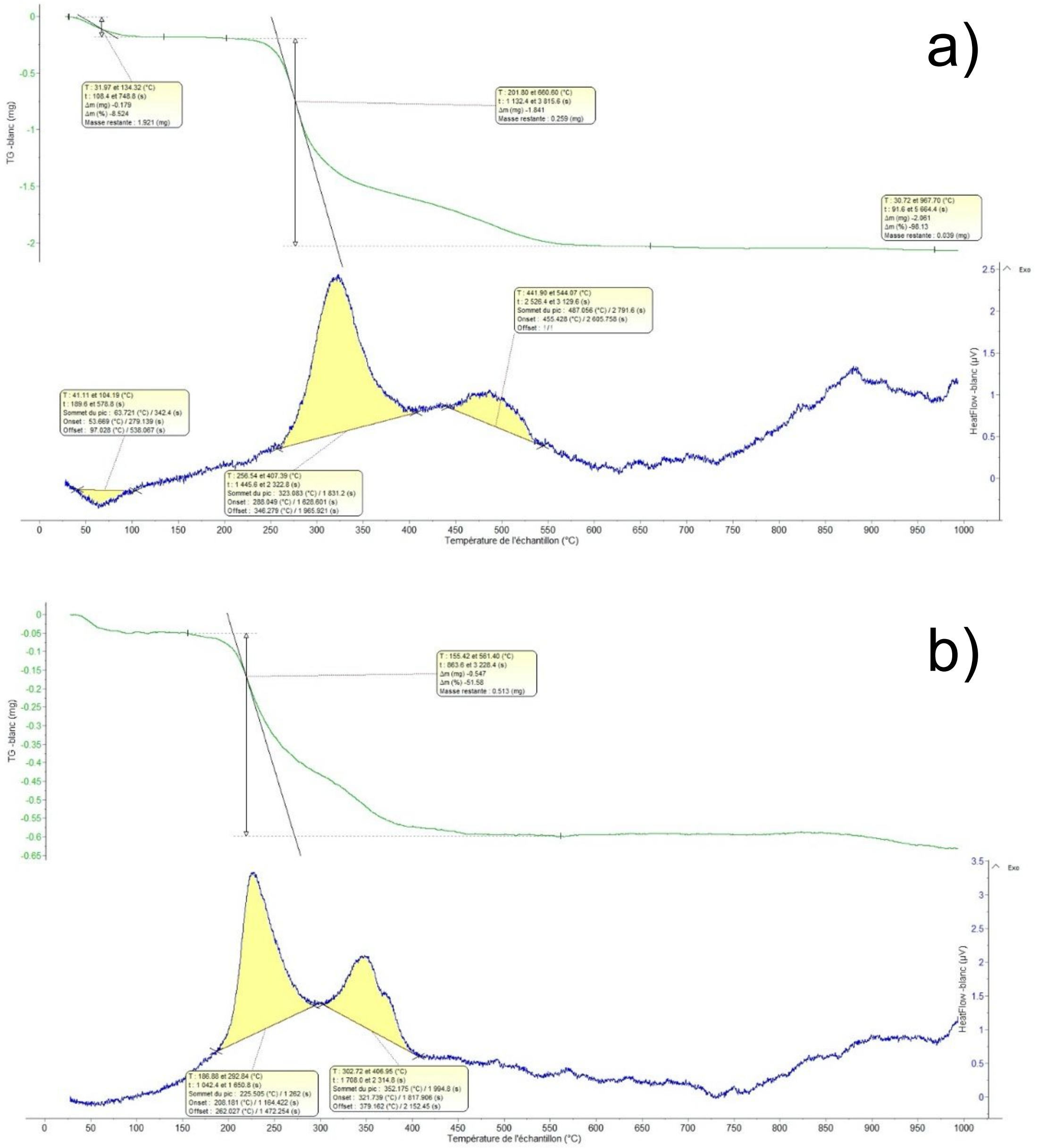

Figure S2: DTG curves of a) native *P. macrosporus* fungus and b) *P. macrosporus*/Au-NPs**.**

Maciel *et al.*

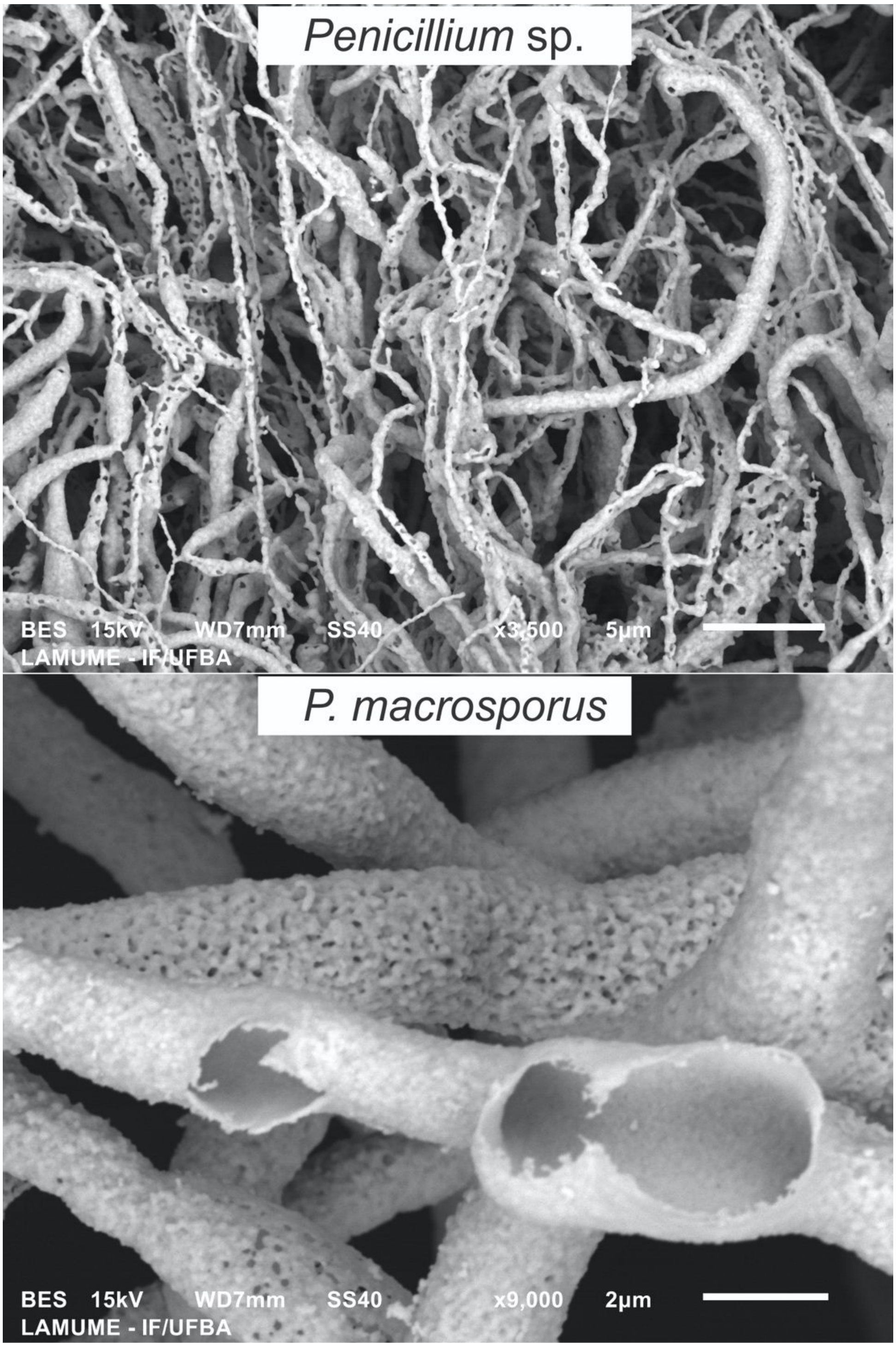

Figure S3: SEM micrographs of mycelium-like microtubes obtained from different fungal species.

Maciel *et al.*

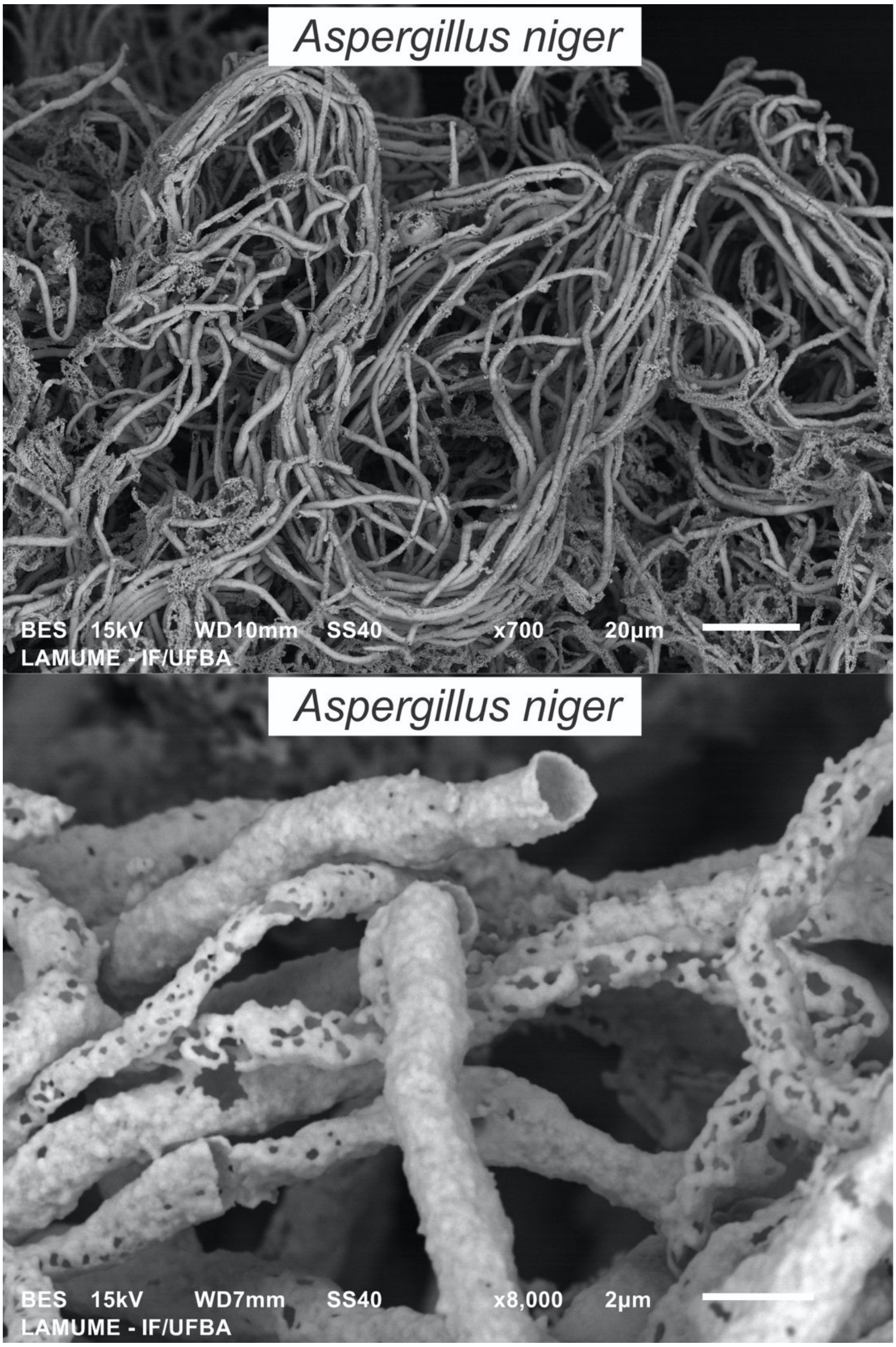

Figure S4: SEM micrographs of mycelium-like microtubes obtained from A. niger/Au biohybrids under different magnifications.

Maciel *et al.*

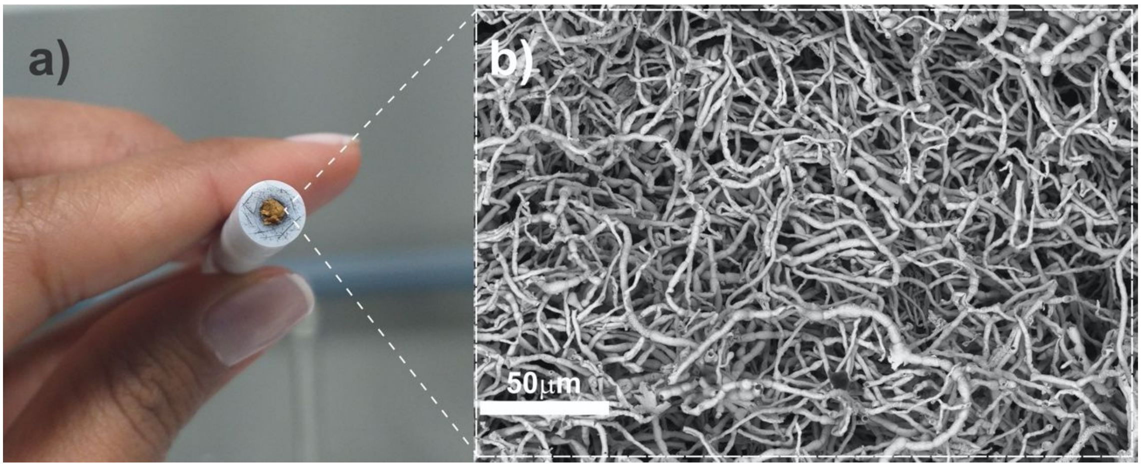

Figure S5: a) Electrode used in electrochemical measurements assembled with the replica of the fungal mycelium. b) SEM micrograph demonstrating the general morphology of mycelium-like gold microtubes.

Maciel *et al.*

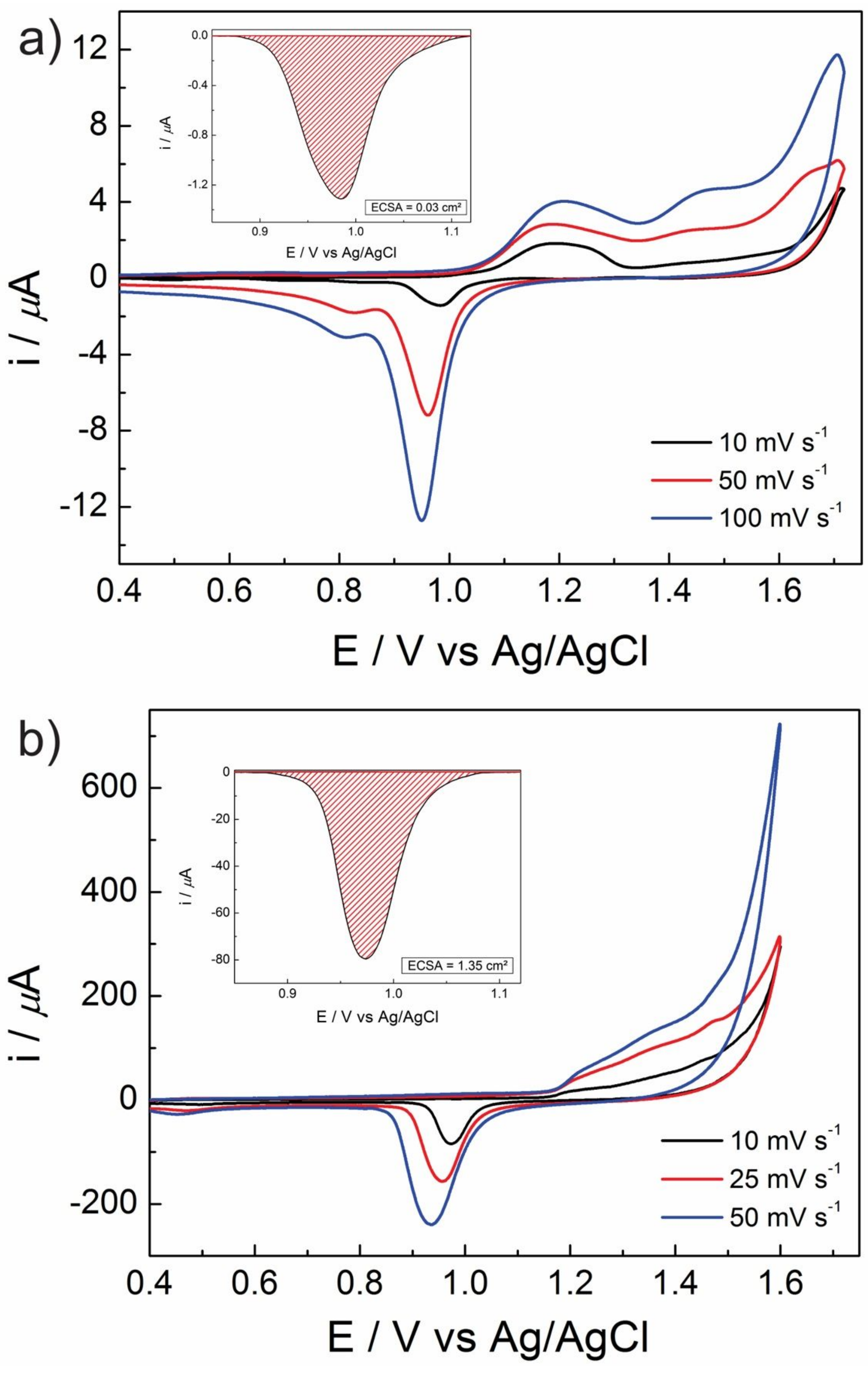

Figure S6: Cyclic voltammograms of (a) bare electrode and (b) mycelium-like microtubes. Inset: Integration of the area of the reduction peak corresponding to the oxide layer.

Maciel *et al.*
